# Heritability patterns in hand osteoarthritis: the role of osteophytes

**DOI:** 10.1186/ar3144

**Published:** 2010-09-28

**Authors:** Mariko L Ishimori, Roy D Altman, Myles J Cohen, Jinrui Cui, Xiuqing Guo, Jerome I Rotter, Michael H Weisman

**Affiliations:** 1 Division of Rheumatology, Cedars-Sinai Medical Center, 8700 Beverly Blvd, B-131, Los Angeles, CA 90048, USA; 2 Division of Rheumatology, The David Geffen School of Medicine at UCLA, 1000 Veteran Ave, #32-59, Los Angeles, CA 90024, USA; 3 Orthopedic Center, Cedars-Sinai Medical Center, 8635 W 3rd Street, 990-W, Los Angeles, CA 90048, USA; 4 Medical Genetics Institute, Cedars-Sinai Medical Center, 116 North Robertson Boulevard, 4th Floor, Los Angeles, CA 90048, USA

## Abstract

**Introduction:**

The objective of the present study was to assess heritability of clinical and radiographic features of hand osteoarthritis (OA) in affected patients and their siblings.

**Methods:**

A convenience sample of patients with clinical and radiographic hand OA and their siblings were evaluated by examination and radiography. Radiographs were scored for hand OA features by radiographic atlas. The heritability of hand OA phenotypes was assessed for clinical and radiographic measures based on anatomic locations and radiographic characteristics. Phenotypic data were transformed to reduce non-normality, if necessary. A variance components approach was used to calculate heritability.

**Results:**

One hundred and thirty-six probands with hand OA and their sibling(s) were enrolled. By anatomic location, the highest heritability was seen with involvement of the first interphalangeal joint (*h*^2 ^= 0.63, *P *= 0.00004), the first carpometacarpal joint (*h*^2 ^= 0.38, *P *= 0.01), the distal interphalangeal joints (*h*^2 ^= 0.36, *P *= 0.02), and the proximal interphalangeal joints (*h*^2 ^= 0.30, *P *= 0.03) with osteophytes. The number and severity of joints with osteophyte involvement was heritable overall (*h*^2 ^= 0.38, *P *= 0.008 for number and *h*^2 ^= 0.35, *P *= 0.01 for severity) and for all interphalangeal joints (*h*^2 ^= 0.42, *P *= 0.004 and *h*^2 ^= 0.33, *P *= 0.02). The severity of carpometacarpal joint involvement was also heritable (*h*^2 ^= 0.53, *P *= 0.0006). Similar results were obtained when the analysis was limited to the Caucasian sample.

**Conclusions:**

In a population with clinical and radiographic hand OA and their siblings, the presence of osteophytes was the most sensitive biomarker for hand OA heritability. Significant heritability was detected for anatomic phenotypes by joint location, severity of joint involvement with osteophytes as well as for overall number and degree of hand OA involvement. These findings are in agreement with the strong genetic predisposition for hand OA reported by others. The results support phenotyping based on severity of osteophytes and a joint-specific approach. More specific phenotypes may hold greater promise in the study of genetics in hand OA.

## Introduction

Osteoarthritis (OA), a polymorphic condition, is a disease of articular cartilage and bone accompanied by subchondral bone thickening, bony outgrowths, and mild chronic synovitis. Hand OA is one of the most common types of OA in developed countries, and is more prevalent in perimenopausal and postmenopausal women. The incidence and prevalence of hand OA has been shown to increase with age; after age 50, women have higher rates than men [1,2]. In contrast to weight-bearing joints such as the knee or the hip, heavy or prolonged use alone has been insufficient to explain the prevalence of hand OA [1-3]. Little is understood regarding the pathogenesis of hand OA, but familial aggregation and heritability studies indicate a significant genetic role in addition to environmental and use-related influences [4-8]. With the high prevalence of hand OA, coupled with the significant direct and indirect costs to the patient and to society, and the lack of effective treatments, understanding the genetic basis for the disease could provide a greater understanding of its pathogenesis and a potential rational basis for treatment or even prevention in the future.

One of the difficulties in addressing hand OA has been the absence of an agreed-upon optimal definition to characterize patients for study. Previous heritability studies have been in different types of patient groups (population-based, twin registry, community volunteers), with most studies using variable definitions of radiographic OA regardless of whether the manifestations of hand OA were clinically present. Most of these studies evaluated the heritability of generalized OA in subjects with involvement of multiple sites, including the hand, knee, hip or spine, but were not specific to hand OA itself. To better understand the specific phenotypes of hand OA, we have undertaken a heritability assessment of our population to see which types of clinical and radiographic features have the greatest heritability. Differential heritability estimates for various traits may contribute to better understanding of the etiology of potential risk factors for hand OA. As part of an ongoing genetic study, subjects with hand OA were recruited for a family-based study and were characterized for clinical and radiographic signs of hand OA.

## Materials and methods

The study subjects were recruited as part of the Genetics of Hand Osteoarthritis Study, aimed at assessing genetic determinants of symptomatic and radiographic hand OA in sibling pairs. The study is based in Los Angeles, CA, USA. Oral and written consent were required from all participants. The protocol was approved by the Institutional Review Board of Cedars-Sinai Medical Center, Los Angeles, CA, USA.

### Recruitment and clinical evaluation

Consecutive, unrelated subjects (probands) with symptomatic (that is, pain or disability), idiopathic (primary) OA of the hand - in the distal interphalangeal joint (DIP) and/or proximal interphalangeal joint (PIP) and/or first carpometacarpal joint (CMC) - were invited to participate as part of a family study. Subjects were recruited by newspaper and electronic advertisement (website, email), mailings in the greater Los Angeles area, and by referral from local rheumatologists and hand surgeons. Study enrollment began in January 2006 and is ongoing.

For inclusion in this study, these subjects must be ≥ 45 years of age, meet American College of Rheumatology clinical classification criteria for OA, and have radiographic evidence of OA in the form of osteophytes scored by a validated atlas in either the trapeziometacarpal (first CMC) joint or at least two DIP or PIP joints [9,10]. Probands were excluded if they had the following conditions: psoriatic arthritis, rheumatoid arthritis, other collagen-vascular disease (for example, systemic lupus erythematosus, scleroderma), history of trauma-induced arthritis, renal failure, known bone disease, calcium pyrophosphate deposition disease, gout, or previous hand joint surgery. Once a proband met the study criteria and participated, we also enrolled any full-blooded living sibling; enrollment of the sibling took place either onsite or offsite anywhere in the USA.

Participating subjects completed a questionnaire reporting demographic data, medical history, medication history, hormone exposure, family history and other sites of OA. All probands and onsite siblings underwent physical examination for signs of hand OA by a single investigator (MLI) at a hospital-based dedicated research facility, along with symptom assessments, hand photography, radiography and blood draw. For offsite siblings, hand X-ray scans were obtained using a standard protocol and blood was drawn in their area of residence. In addition, all probands completed the Australian/Canadian OA Hand Index using a five-point Likert-scale format that contains 15 questions divided into three areas: pain (five questions), stiffness (one question) and physical function (nine questions).

A posterior radiograph of both hands was obtained and blood was collected for genetic analysis. Blinded hand radiographs were scored by a single reader (RDA), without knowledge of the study subject's other data. Radiographs were scored by an atlas for osteophytes (0 to 3), sclerosis (0 to 3), joint space narrowing (0 to 3), subchondral cysts (presence/absence) and erosions (presence/absence) [10]. Randomized re-reading of radiographs for intra-reader reliability demonstrated an intraclass correlation > 0.90 for both the joint score and the joint count. Finally, the presence of radiographic osteophytes in 10 selected joints (the CMC, PIP, DIP and first interphalangeal (IP) joints) was used to generate two sum scores: the total number of joints with any osteophytic change ranging from 0 to 20 (Total Count); and the sum of osteophyte scores (range 0 to 3) from each of the 10 affected joints in each hand involved, ranging from 0 to 60 (Total Score). The Total Count and the Total Score were used as a surrogate for radiographic disease severity.

The frequency and heritability of radiographic hand OA phenotypes were assessed. The phenotypes included overall hand OA involvement as measured by the Total Count and the Total Score, and radiographically assessed phenotypes based on anatomic locations of joint groups (DIP ± PIP ± CMC) and radiographic characteristics, such as joint space narrowing, osteophytes, or subchondral erosions. The scores for each type of radiographic feature were summed for the 10 joints in each hand on a quantitative scale, and a total score was obtained ranging from 0 to 60.

### Heritability and correlation analyses

Subjects and their siblings were assessed for the agreement of specific phenotypic traits within their family. Phenotype traits were transformed (log or square-root) to reduce non-normality if necessary. The variation in each trait was assumed to be a linear combination of covariate effects, genetic effect and residual variability. A variance components approach was used in which the total phenotypic variance (σ^2^_p_) was partitioned into the variance due to the additive effects of genes (σ^2^_G_) and the variance due to environmental effects (σ^2^_E_). The genetic effect was assumed to be independent and normally distributed with zero mean and variance of σ^2^_G_. The heritability (h^2^) of each trait, which quantifies the extent to which genetic variation contributed to the expression of the specific phenotypes, independent of disease prevalence, was calculated by the genetic variance divided by the total phenotypic variance. Age, gender and body mass index were included as covariates in the analysis to adjust for potential confounding effect. The heritability was calculated using SOLAR software (Southwest Foundation of Biomedical Research, San Antonio, TX, USA) [11]. We performed these analyses in the sample as a whole and then, as a sensitivity analysis, repeated them restricting the analysis to Caucasians.

## Results

One hundred and thirty-six families consisting of probands and at least one sibling were enrolled. Of these families, 150 siblings were enrolled and 77 of the siblings were enrolled onsite with a complete examination of the hands. As expected, the majority of the probands were women. Among the siblings, 32% (48/150) were men. Most participants were over 60 years of age, were Caucasian, had disease for over 10 years, and were only mildly overweight (Table 1).

**Table 1:** Characteristics of hand osteoarthritis probands and families

Proband sex (male:female)	13 (9.6%):123 (90.4%)
Average age of probands (years)	65.5 ± 8.51
Average age of siblings (years)	63.8 ± 9.10
Ethnic/racial breakdown of families	
Caucasian	121 (89%)
Asian	8 (5.9%)
Hispanic	6 (4.4%)
Other	1 (0.7%)
Average number of siblings/family	1.1 (1 to 3)
Average duration of hand osteoarthritis in probands	11.7 years
Average body mass index of probands	25.8 ± 4.54
Average body mass index of siblings	26.6 ± 5.30

Data presented as number (percentage), mean ± standard deviation, or mean (range).

After adjusting for age, gender and body mass index, our results revealed that the greatest statistical significance for heritability was for radiographic osteophytes at anatomic locations in the interphalangeal joints of probands and siblings, with the highest heritability seen for the involvement of the IP joint (*h*^2 ^= 0.63, *P *= 0.00004) and the CMC joint (*h*^2 ^= 0.38, *P *= 0.01), but with significant heritability estimates for the DIP joints (*h*^2 ^= 0.36, *P *= 0.02) and the PIP joints (*h*^2 ^= 0.30, *P *= 0.03) (Table 2). When subjects were assessed for the total number of any IP joints (DIP/IP/PIP count) involved, which could include the DIP and/or IP and/or PIP joints in probands and their siblings, the heritability estimate was 0.42 (*P *= 0.004); for the Total Count, which also included CMC joints, the heritability estimate was 0.38 (*P *= 0.008).

**Table 2:** Heritability estimates for radiographic phenotypes, after adjustment for age, gender and body mass index

	All subjects		Caucasians only	
		
Phenotype	Heritability estimate (SE)	*P *value	Heritability estimate (SE)	*P *value
First IP count	**0.63 (0.15)**	0.00004	**0.70 (0.15)**	0.00001
DIP count	**0.36 (0.1643)**	0.02	**0.36 (0.17)**	0.019
PIP count^a^	**0.30 (0.16)**	0.03	**0.28 (0.17)**	0.04
DIP/IP/PIP count^a^	**0.42 (0.16)**	0.004	**0.41 (0.16)**	0.006
CMC count	**0.38 (0.17)**	0.01	**0.46 (0.17)**	0.005
Total count^a^	**0.38 (0.16)**	0.008	**0.38 (0.16)**	0.01
First IP score^a^	**0.84 (0.14)**	0.0000001	**0.89 (0.14)**	2.70 × 10^-9^
DIP score^a^	0.18 (0.16)	0.12	0.16 (0.17)	0.17
PIP score^a^	0.16 (0.15)	0.15	0.14 (0.16)	0.18
DIP/IP/PIP score^a^	**0.33 (0.16)**	0.02	**0.33 (0.16)**	0.02
CMC score	**0.53 (0.16)**	0.0006	**0.63 (0.16)**	0.0001
Total score^a^	**0.35 (0.16)**	0.01	**0.36 (0.16)**	0.01
Subchondral cysts^a^	0.17 (0.15)	0.13	0.13 (0.16)	0.20
Joint subluxation^a^	0.27 (0.17)	0.06	0.19 (0.19)	0.15
Joint space narrowing^a^	0.24 (0.16)	0.06	0.18 (0.17)	0.14
Subchondral sclerosis^a^	0.17 (0.16)	0.14	0.15 (0.17)	0.18
Erosions^a^	0.12 (0.14)	0.20	0.13 (0.15)	0.20
Joint deformity^a^	0.31 (0.20)	0.06	0.15 (0.20)	0.24
Heberden's nodes	0.36 (0.22)	0.07	0.21 (0.23)	0.19

CMC, carpometacarpal joint; DIP, distal interphalangeal joint; IP, interphalangeal joint; PIP, proximal interphalangeal joint; SE, standard error. ^a^Transformed by square root. Statistically significant results are represented in bold.

In addition to the number of specific joints involved, the severity of osteophytes was also scored for each involved joint, and heritability estimates were significant for the IP score, the DIP/IP/PIP score, the CMC score and the Total Score. Heritability estimates for anatomic phenotypes including the Total Count and the Total Score emphasize the significant heritability of all joint counts and several scores, particularly the CMC score (Table 2). When the Caucasian sample of patients was analyzed separately, the heritability estimates of significance were maintained (see Table 2).

With regard to radiographic features of joint space narrowing and subluxation, there was a trend towards significance; but for other features such as subchondral sclerosis, subchondral cysts, and erosions, the heritability was nonsignificant. The overall levels of osteophyte formation that were used to generate the sums of Total Score and Total Count were both heritable at 0.35 and 0.38, respectively (Table 2). Clinical features of Heberden's nodes and joint deformity showed a trend towards significance.

## Discussion

In our population of 136 subjects with clinical and radiographic hand OA and 150 of their siblings, osteophyte involvement was the most heritable feature compared with other clinical and radiographic phenotypes. This result was observed for the presence of osteophytes in the DIP, PIP, and IP joints as well as for total IP joint involvement, and the Total Count as analyzed in the overall group, as well as when analyses were restricted to the Caucasian sample. Moreover, when the severity of osteophyte involvement in these joint groups was scored, heritability estimates were significant for the IP score, the DIP/IP/PIP score, the CMC score, and the Total Score. Other radiographic and clinical features displayed a trend toward significance, such as joint space narrowing, and joint subluxation on radiographs, and joint deformity and Heberden's nodes on examination.

The main goal of the present study was an attempt to demonstrate which clinical and/or radiographic phenotypes of hand OA might have the highest heritability in a condition known to have a genetic risk. The highest heritability estimates were observed for the first IP count and score (*h*^2 ^= 0.63 and *h*^2 ^= 0.84, respectively), the CMC score (*h*^2 ^= 0.53) and the DIP/IP/PIP count (*h*^2 ^= 0.42), which were based on the presence of osteophytes. We also observed that disease severity as assessed by the presence of osteophytes, a classic hand OA feature, was heritable, with greater involvement in the number of joints and severity per joint seen in sibling pairs. This finding was observed despite the fact that siblings were invited to participate regardless of their hand OA status. The heritability of these individual phenotypes supports the argument that phenotyping based on severity of osteophytes and location of disease is an important consideration for a genetic study.

The influence of heredity in hand OA has been observed and studied in a variety of ways, including the assessment of relative risk in siblings, aggregation in families, and disease concordance in twins. Stecher in 1941 noted a hereditary disposition for hand OA expression, with a twofold excess of disease in mothers and a threefold excess in sisters of patients with Heberden's nodes compared with unrelated controls [8]. Kellgren and colleagues noted similar estimates [12]. A classic twin study of hand OA in women showed a higher concordance of Heberden's nodes and radiographic disease in monozygotic twins when compared with dizygotic twins with both hand and knee disease [7].

Further, hand OA disease heritability has been assessed in population-based studies looking at radiographic OA [4-6,13]. In the Framingham Offspring Study, sibling-sibling and parent-offspring correlations were found for the OA count (number of hand and knee joints affected with OA), with higher correlations among female pairs compared with male pairs [5]. Similarly, a population-based study in the Netherlands revealed a heritability estimate of 0.56 for hand OA in a group of patients with hand OA and degenerative spine disease [4]. The Baltimore Longitudinal Study of Aging also reported a familial aggregation of hand OA [6]. More recently, Jonsson and colleagues noted that sisters of patients with interphalangeal joint and CMC joint OA have relative risks of 5.0 and 6.9, respectively, to develop OA in the same joint [13].

A genetic influence on radiographic OA has been noted to be specific to joint groups and not for OA as a whole, particularly in women [14]. The results in our population revealed heritability estimates to be statistically significant for joint groups (DIP, IP and PIP) and for severity of involvement at interphalangeal joints and the CMC joints. This is consistent with observations in studies by Hirsch and colleagues and Jonsson and colleagues, which were both population-based studies [6,13]. Other clinical and radiographic phenotypes possessed a trend towards significance in the present study, but statistical significance was not attained in the current sample size.

There are several limitations to our study (imposed by its design) that could impact on its interpretation and generalizability. Our collection is a convenience sample and the current analysis focused on 136 completed families. There are other families for whom the sibling enrollment has not yet been completed, and the study enrollment continues. In addition, the enrollment of any available sibling may lead to some selection bias, as those siblings affected by hand OA may be more likely to participate. Whenever possible, every attempt was made to enroll as many siblings per family as possible - although given the average age of the subjects (65.5 years), in some instances certain siblings were either too ill or deceased and could not participate. At the time of this study, the completed families represented 74.7% of probands with available siblings.

Ultimately, this cohort will be studied to assess for genetic associations, for which a bias towards affected sibling participation may potentially be an advantage, as an enriched population for genetic study. For some phenotypes with an observed trend toward significance, a larger sample size will need to be studied. The family structure is also simplified, with analysis of siblings only, which may limit the interpretability of variance component analysis and the possible contribution of a common environment is therefore difficult to evaluate. In this analytic situation, the heritability estimate is the upper bound and includes the possible common environment. In most cases, due to the age of the study patients and their siblings, very few parents are living, and the offspring may be too young to enroll as they may not as yet have expression of hand OA. Nevertheless, the subjects who have been enrolled have been carefully phenotyped radiographically.

For the clinical characteristics contributed by a physical examination, the sample size is limited to the 77 families in whom enrollment was completed onsite. This does limit the sample size for these clinical phenotypes. As many study subjects were referred from rheumatology or hand surgery practices, our patients may also possess a more severe form of hand OA when compared with the general, community-based population. The study population was predominantly Caucasian women, of whom 40% were of Ashkenazi Jewish descent, which may potentially lead to bias. For genetic analyses, however, more severe forms of disease expression and more genetically homogeneous populations may identify groups with greater genetic predisposition.

There are inherent limitations to heritability studies. While a higher heritability suggests that the phenotype is a good predictor of genotype, it does not automatically mean that knowing the genotype determines the phenotype; other factors may come into play. Additional limitations to heritability analysis include the following: heritability is a measure of correlation and does not identify genes; there is no direct correlation between higher heritability and genes of larger effect; heritability estimates can vary from one population to another; heritability does not preclude environmental influences on a trait; and in heritability analyses limited to siblings, the heritability estimate should be considered an upper bound, confounded, in part, by the common sibling environment. Nevertheless, assessment of heritability is considered an appropriate first step before one proceeds in efforts to identify genes.

In an attempt to analyze heritability, the subdivision of the population into the phenotypes of joint location and clinical and radiographic features may lead to smaller sample sizes for some groups, which may limit the utility of a given analysis. With the dearth of information available regarding carefully characterized phenotypes in hand OA in the literature, however, even an analysis of the smaller sample will help guide the phenotypes upon which to focus future genetic analysis. Genetic studies of hand OA to date have used clinical phenotypes, radiographic phenotypes or anatomic location for genetic studies. It is possible that the dissimilarity of analyzed OA features has lead to a difficulty in reproducing genotype associations in different studies [15,16]. The plan for the present study going forward is therefore to characterize the population for clinical, radiographic and anatomic phenotypes to determine those features showing the greatest heritability.

## Conclusions

In our study of 136 probands with clinical and radiographic hand OA and of 150 siblings, the presence of osteophytes was the most heritable feature compared with other clinical and radiographic phenotypes. Significant heritability was detected for radiographic involvement with osteophytes overall and by joint location and severity, with the highest heritability estimates for the involvement of the first IP and CMC. These findings are in agreement with the strong genetic predisposition for hand OA reported by others. It is possible to hypothesize that there may be a heritable component to hand OA that contributes to the concept of osteophyte load as well as the site of affected joints. Our analysis also supports phenotyping on the basis of osteophyte severity and a joint-specific approach, as more specific phenotypes may hold greater promise in the study of the genetic basis of hand OA.

## Abbreviations

CMC: carpometacarpal joint; DIP: distal interphalangeal joint; IP: interphalangeal joint; OA: osteoarthritis; PIP: proximal interphalangeal joint.

## Competing interests

The authors declare that they have no competing interests.

## Authors' contributions

MLI contributed to conception and design, acquisition of data, analysis and interpretation of data, drafting of the article and final approval of the submitted manuscript. RDA contributed to the conception and design of the study, the acquisition and interpretation of data, revision for important intellectual content and final approval of the manuscript. MJC contributed to the provision of patients, acquisition of data, revision for important intellectual content and final approval of the manuscript. JC contributed to analysis and interpretation of data, revision for important intellectual content and final approval of the manuscript. XG contributed to the design of the study, analysis and interpretation of data, revision for important intellectual content and final approval of the manuscript. JIR contributed to conception and design of the study, revision for important intellectual content and final approval of the manuscript. MHW contributed to conception and design of study, revision for important intellectual content and final approval of the manuscript. MLI and MHW take responsibility for the integrity of the research and manuscript, from inception to finished article.
